# When inclusive leadership meets supervisory interaction pressure: explaining innovative behavior among public employees

**DOI:** 10.3389/fpsyg.2026.1960198

**Published:** 2026-09-29

**Authors:** Xiaoting Zhong, Guoxian Bao

**Affiliations:** 1 School of Management, Lanzhou University, Lanzhou, China; 2 China Research Center for Chinese Government Performance Management, Lanzhou University, Lanzhou, China; 3 School of Management, Northwest Normal University, Lanzhou, China

**Keywords:** civil servants, inclusive leadership, innovative behavior, psychological contract, public service motivation, supervisory interaction pressure

## Abstract

**Introduction:**

Public organizations increasingly depend on employees to identify service failures and implement improvements, yet immediate supervisors combine developmental support with evaluative authority. Drawing on job demands-resources theory and integrating psychological contract and public service motivation research, this study examines how inclusive leadership is associated with innovative behavior and when supervisory interaction pressure constrains this relationship.

**Methods:**

Cross-sectional survey data were collected from 4,511 tax civil servants across a provincial tax administration system in China and analyzed using confirmatory factor analysis and moderated sequential mediation analyses.

**Results:**

Inclusive leadership was positively associated with innovative behavior directly, and the observed indirect association was consistent with the proposed ordering of psychological contract and public service motivation. Supervisory interaction pressure weakened the positive relationships between inclusive leadership and both psychological contract and innovative behavior. However, the moderated mediation tests did not provide sufficient statistical evidence that supervisory interaction pressure significantly weakened the indirect or ordered sequential indirect relationships. Inclusive leadership nevertheless remained positively associated with both outcomes at higher levels of supervisory interaction pressure, indicating that interaction pressure constrained rather than eliminated its benefits.

**Discussion:**

The study contributes to public personnel management research by showing how the relational conditions surrounding supervisory interaction shape the use and interpretation of inclusive leadership as a job resource. It further explains how supportive supervisory conduct is associated with perceived reciprocity, public service motivation, and innovative behavior.

## 1 Introduction

Public organizations increasingly rely on employees to identify service failures, adapt administrative processes, and respond to changing social, technological, and policy conditions (De Vries et al., 2016; Criado et al., 2025). At the employee level, innovation involves generating ideas, gaining support, and implementing improvements in everyday work practices (Scott and Bruce, 1994; Janssen, 2000). Employees who work close to administrative processes and service delivery are well placed to identify operational bottlenecks and shifting citizen needs because local knowledge and discretion shape policy implementation (Tummers and Bekkers, 2014). Yet this proximity also makes innovation difficult. Employees who question established routines or advance uncertain alternatives must secure organizational support while remaining accountable to formal mandates and procedures (Hupe and Hill, 2007). Public-sector innovation must therefore balance improvement with risk, compliance, and procedural reliability (Brown and Osborne, 2013). This tension places immediate supervisors at the center of a distinctive public personnel management challenge.

Immediate supervisors occupy this central position because employees encounter organizational support and formal authority through the same relationship. Employees often interpret supervisory treatment as evidence of organizational regard, while public-sector line managers translate formal personnel policies into everyday practice (Eisenberger et al., 2002; Op De Beeck et al., 2017). Inclusive leadership represents the supportive side of this role. Through openness, accessibility, and responsiveness, inclusive supervisors invite employee input and lower the interpersonal risks of raising concerns or tentative ideas (Nembhard and Edmondson, 2006; Carmeli et al., 2010; Alang et al., 2022). Yet supervisors also set expectations, monitor performance, provide evaluative feedback, and enforce formal standards (Lin and Kellough, 2019). Public performance management thus combines employee development with accountability within routine supervisor-employee interactions (Hill and Plimmer, 2024). Employees may consequently perceive a supervisor as inclusive while still experiencing direct encounters with that supervisor as psychologically pressuring. We use supervisory interaction pressure (SIP) to refer narrowly to the psychological pressure employees experience during direct interaction with their immediate supervisor. SIP therefore concerns the experienced pressure of the supervisory interaction itself rather than generalized performance pressure or the broader set of demands associated with supervision.

These two literatures portray different sides of the same supervisory relationship. Inclusive leadership research treats openness, accessibility, and responsiveness as relational resources that lower interpersonal barriers and support employee voice and innovation (Nembhard and Edmondson, 2006; Carmeli et al., 2010; Alang et al., 2022). Public performance-management research, by contrast, emphasizes supervisors' simultaneous responsibilities for employee development, performance evaluation, and organizational accountability (Hill and Plimmer, 2024). The unresolved issue is not whether supportive and demanding features of supervision can coexist, but how employees use and interpret inclusive leadership when direct interaction with the same authority figure feels psychologically pressuring. Such pressure may make supervisory support more difficult to use and may also alter the relational meaning employees assign to inclusive conduct. The central question is therefore when supervisory interaction pressure constrains the positive association between inclusive leadership and innovative behavior and how this boundary condition relates to employees' relational and public-service-oriented responses.

Explaining these associations requires distinguishing employees' relational interpretation of inclusive leadership from the public-service orientation associated with discretionary effort. Psychological contract theory addresses the first element. Because supervisors act as organizational agents, employees may interpret openness, accessibility, and responsiveness as evidence of recognition and reciprocal organizational support (Eisenberger et al., 2002; Dabos and Rousseau, 2004). Public service motivation captures a distinct motivational orientation toward public interests and socially valued outcomes (Vandenabeele, 2007). Although PSM has relatively enduring foundations, leadership and organizational conditions can shape its workplace expression (Hameduddin and Engbers, 2022). Therefore, we theorize an ordered relational-to-public-service association in which favorable psychological contract perceptions are associated with stronger expression of public service motivation, which in turn is associated with innovative behavior (Miao et al., 2018).

Job demands–resources theory provides the overarching logic for examining how supervisory resources operate under relational demands. JD-R research recognizes that job demands and resources can coexist and jointly shape employee functioning, while leadership can influence both the work conditions public servants experience and the way those conditions affect them (Bakker and Demerouti, 2017; Tummers and Bakker, 2021). Building on this perspective, we focus on a more specific relational contingency: whether psychological pressure experienced in direct interaction with a supervisor conditions the value of inclusive conduct from that same supervisor. Such pressure may affect both public servants' use of supervisory support and the relational meaning they assign to inclusive leadership.

We examine these relationships among tax civil servants in a provincial Chinese tax administration. Tax administration provides a theoretically informative setting because public servants must reconcile enforcement and regulatory responsibilities with taxpayer service while operating under formal rules, performance appraisal, and hierarchical accountability (Wang et al., 2019). Innovation is therefore necessary but institutionally bounded: public servants may identify opportunities to improve administrative processes or taxpayer services, yet acting on those opportunities often requires coordination, authorization, and consistency with formal requirements (Criado et al., 2025; Brown and Osborne, 2013). Immediate supervisors are especially consequential under these conditions because they provide information and implementation support while also communicating expectations, monitoring work, and exercising evaluative authority (Schwarz et al., 2020). Therefore, this setting makes the relational boundary condition examined here particularly visible: supportive supervisory conduct and psychologically pressuring supervisory interaction can be experienced within the same hierarchical relationship.

This study makes two contributions to public personnel management research. First, it develops a relationally contingent account of inclusive supervision. Building on the established JD-R premise that demands and resources jointly shape employee functioning, we specify how supervisory interaction pressure may condition the value of inclusive leadership within the same authority relationship. The model distinguishes two ways in which this relational contingency may operate: interaction pressure may make supervisory resources more difficult to use, and it may weaken the credibility of inclusive conduct as a signal of organizational recognition and reciprocity. This shifts attention from whether supervisory resources and demands coexist to how the relational conditions surrounding a supervisor shape employees' ability to benefit from supportive leadership. Second, it develops a relational-to-public-service account of the association between inclusive leadership and innovative behavior. Psychological contract perceptions capture employees' assessment of mutuality and reciprocity in the employment relationship, whereas PSM captures the motivational orientation that connects discretionary effort with public purposes (Dabos and Rousseau, 2004; Vandenabeele, 2007). By theoretically ordering these constructs, the model links employees' interpretation of inclusive supervisory conduct with a public-service orientation toward innovative behavior. This ordering is conceptual rather than evidence of temporal causality.

## 2 Theoretical framework and hypotheses

### 2.1 An integrative framework of supervisory resources, relational interpretation, and public-service motivation

Figure 1 integrates job demands-resources theory, psychological contract theory, and public service motivation research to explain how inclusive leadership is associated with innovative behavior and when this relationship is constrained. JD-R theory provides the overarching resource-demand logic by distinguishing job demands from job resources and explaining how their combination shapes employee strain, motivation, and work functioning (Demerouti et al., 2001; Bakker and Demerouti, 2017). It allows job demands and resources to operate jointly and recognizes leadership as relevant to the work conditions through which employees experience demands and resources (Bakker and Demerouti, 2017; Tummers and Bakker, 2021; Bakker et al., 2023). Within this framework, inclusive leadership represents a supervisory resource by providing assistance, access, and opportunities to participate, whereas supervisory interaction pressure represents a relationship-specific demand associated with psychologically pressuring direct contact with the immediate supervisor. Psychological contract theory specifies how employees interpret inclusive conduct as evidence of organizational recognition and reciprocity, while PSM explains why they may direct discretionary effort toward improvements that serve organizational and public purposes (Dabos and Rousseau, 2004; Vandenabeele, 2007).

**Figure 1:**
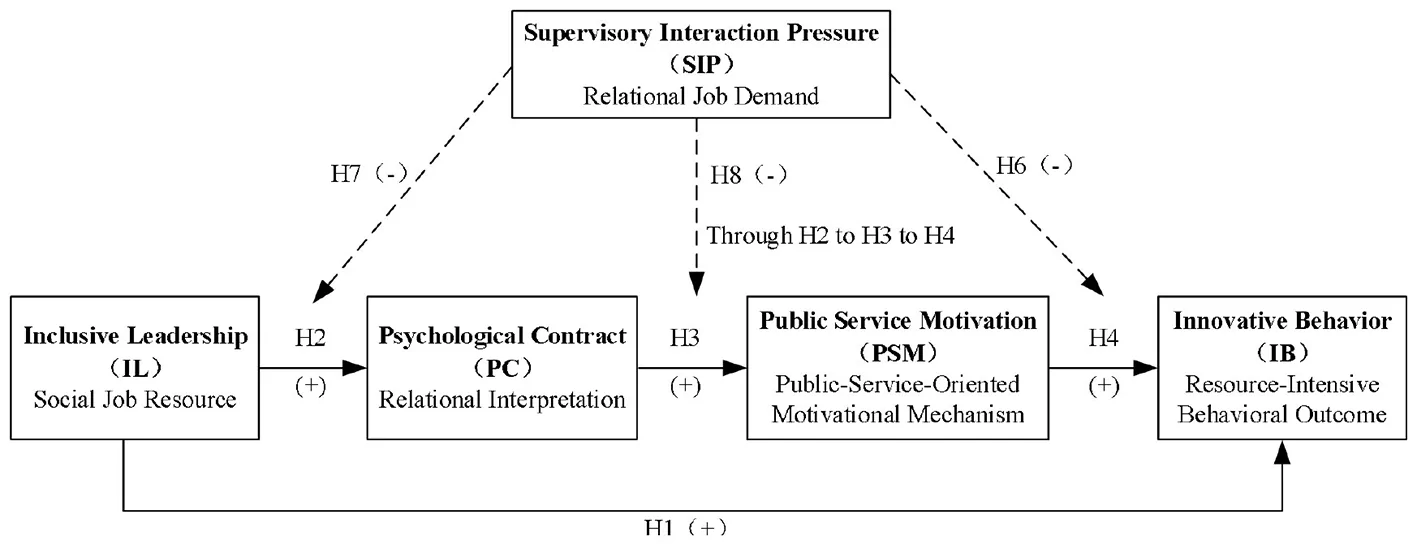
Research model.

Importantly, the model locates the resource and demand within the same supervisory relationship. Public-sector supervisors support employee development while also monitoring performance and enforcing accountability (Op De Beeck et al., 2017; Lin and Kellough, 2019; Hill and Plimmer, 2024). When direct interaction with such a supervisor feels psychologically pressuring, employees may find the support associated with inclusive leadership more difficult to use. Because employees also treat supervisors as organizational representatives, pressure attached to the same relationship may make inclusive conduct a less credible signal of organizational recognition and reciprocity. Supervisory interaction pressure is therefore theorized to condition both employees' use of inclusive leadership as a resource and their interpretation of its relational meaning.

### 2.2 Inclusive leadership as a supervisory resource

Within the proposed framework, inclusive leadership is the supervisory resource that initiates the relational and behavioral pathway. We define it as observable conduct through which immediate supervisors invite employee input, remain accessible, and respond constructively to employees' ideas and concerns (Nembhard and Edmondson, 2006; Carmeli et al., 2010). This interactional definition focuses on employees' day-to-day experience of a specific supervisor rather than broader inclusion climates or diversity-management systems. The distinction is important in public organizations, where employees often depend on immediate supervisors for information, assistance, and opportunities to advance improvements. Public-sector research also links inclusive leadership with employee voice and workplace inclusion (Alang et al., 2022). By lowering interpersonal barriers and increasing access to supervisory support, inclusive leadership provides resources that employees can use to develop and implement innovative ideas.

These supervisory resources match the demands of innovative behavior. Generating ideas requires employees to question established routines, promoting them entails rejection and reputational risks, and implementation often depends on information, authorization, and organizational support (Scott and Bruce, 1994; Yuan and Woodman, 2010; Wynen et al., 2020). Inclusive supervisors reduce these barriers by inviting employee input, remaining accessible when assistance is needed, and responding constructively to tentative proposals (Nembhard and Edmondson, 2006; Carmeli et al., 2010). Such support is particularly important in public organizations, where employees must advance improvements through formal mandates and hierarchical approval. Evidence from public-sector organizations indicates that empowering and supportive management helps employees identify service improvements and engage in innovative behavior (Miao et al., 2018; Fernandez and Moldogaziev, 2013). Inclusive leadership should therefore help public employees translate their operational knowledge into innovative action. Recent evidence further supports this relationship in public-sector settings. AlMunthiri et al. (2024) found that inclusive leadership was positively associated with innovative behavior among public-sector employees, both directly and indirectly through work engagement. Complementing this evidence, He and Li (2026) showed that inclusive leadership can foster employee innovative behavior through positive emotions and creative self-efficacy.

Based on the preceding arguments, we propose the following hypothesis:

**Hypothesis 1 (H1):** Inclusive leadership is positively associated with public servants' innovative behavior.

Beyond providing resources for innovation, inclusive leadership can shape employees' psychological contract with the organization. A psychological contract reflects employees' beliefs about the reciprocal obligations between themselves and their employer (Rousseau, 1989). Because organizations act through managers, employees often read the conduct of immediate supervisors as evidence of organizational intent (Eisenberger et al., 2002; Shoss et al., 2012). This role is especially important in public organizations, where line managers translate formal personnel policies into daily decisions about feedback, support, and access to opportunities (Op De Beeck et al., 2017). Employees may therefore interpret inclusive conduct as evidence of how the organization values and treats them. This interpretation is consistent with recent psychological contract research emphasizing that employees read managerial and organizational actions as signals of the broader exchange relationship. Diehl et al. (2026) further suggest that psychological contracts may include purpose- and value-related expectations, which is especially relevant in public-service settings.

Different aspects of inclusive leadership convey related but distinct messages. When supervisors invite input, they signal that employee perspectives are legitimate organizational contributions. Accessibility shows that help is available when difficulties arise, while constructive responsiveness indicates that concerns and proposals will receive serious consideration (Nembhard and Edmondson, 2006; Carmeli et al., 2010; Alang et al., 2022). Employees also attend to what happens after they speak. Managerial endorsement and follow-through determine whether participation appears genuinely valued or merely symbolic (Burris, 2012).

These signals become more credible when inclusive conduct is sustained across daily interactions and reflected in subsequent decisions. Consistent managerial practices help employees infer organizational intentions and clarify what each side owes the other (Rousseau, 2001; Nishii et al., 2008). Such consistency is particularly consequential in public personnel systems because employees experience formal arrangements through the way line managers implement them (Op De Beeck et al., 2017). When supervisors repeatedly invite contributions, provide assistance, and respond constructively, employees have stronger reason to believe that the organization recognizes their effort and will fulfill reciprocal obligations. Inclusive leadership should therefore strengthen employees' psychological contract with the organization.

Based on the preceding arguments, we propose the following hypothesis:

**Hypothesis 2 (H2):** Inclusive leadership is positively associated with public servants' psychological contract.

### 2.3 From relational reciprocity to public-service-oriented innovation

Having established inclusive leadership as both a supervisory resource and a signal of organizational treatment, we now turn to the process that carries its effects forward. Psychological contract and PSM capture distinct but complementary aspects of public servants' work experience. Psychological contract concerns perceived mutual obligations and reciprocity within the current employment relationship (Rousseau, 2001; Kiefer et al., 2022), whereas PSM reflects a broader motivational orientation toward serving public interests and socially valued outcomes (Perry et al., 2010; Piatak et al., 2020). Because inclusive supervisory conduct is interpreted within the ongoing employment relationship, psychological contract provides a relatively proximal relational link between leadership and motivation. PSM, by contrast, captures the public-service orientation through which public servants connect discretionary effort with public purposes. On this basis, we position psychological contract before PSM in the proposed pathway.

#### 2.3.1 Psychological contract and public service motivation

PSM reflects a relatively enduring motivation to serve public interests and contribute to socially valued outcomes (Perry et al., 2010; Perry and Wise, 1990; Perry, 2014). It may develop before employees enter a particular organization and can influence their employment preferences and sector choices (Vandenabeele, 2008; Holt, 2018). Public employees may therefore bring established public service motives into the employment relationship.

Enduring motives are not fixed, however. Employees' experiences after organizational entry can sustain, strengthen, or weaken their PSM. Longitudinal evidence shows that PSM can change through employment and socialization, while leadership and public HRM practices shape the importance employees attach to public service in their work (Hameduddin and Engbers, 2022; Piatak et al., 2020; Kjeldsen and Jacobsen, 2013). Leadership that connects employees' roles with a compelling public mission has likewise been associated with stronger PSM (Wright et al., 2012). PSM can thus have enduring roots while still varying in strength and salience across organizational settings.

Psychological contract provides one basis for such variation. It captures employees' beliefs about the obligations that they and the organization owe one another, including whether their respective contributions are mutual and reciprocal (Dabos and Rousseau, 2004). When employees believe that the organization recognizes their contributions and will honor corresponding obligations, public-oriented effort is less likely to feel like a one-sided commitment. Public-sector research shows that employees' beliefs about employer obligations and inducements influence helping, advocacy, and other discretionary contributions through reciprocal exchange processes (Coyle-Shapiro and Kessler, 2000).

This sense of reciprocity also shapes how employees connect their public service values with their current organizational role. An organization that recognizes employee contributions and meets its obligations offers a more credible setting in which to pursue public purposes. Under these conditions, public service is more likely to remain personally important and central to employees' motivation at work. Consistent with this argument, reciprocity norms are associated with the norm-based and affective foundations of PSM, while mission-oriented leadership can strengthen employees' motivation to serve the public through their organization (Wright et al., 2012; Neumann, 2019).

Based on the preceding arguments, we propose the following hypothesis:

**Hypothesis 3 (H3):** Public servants' psychological contract is positively associated with their public service motivation.

#### 2.3.2 Public service motivation and innovative behavior

PSM should direct discretionary effort toward innovative behavior. Generating, promoting, and implementing new ideas requires employees to move beyond routine responsibilities and invest sustained cognitive and interpersonal effort (Scott and Bruce, 1994). Idea promotion also exposes employees to rejection and reputational risks, while implementation may provide uncertain or delayed personal returns (Janssen, 2000; Yuan and Woodman, 2010). PSM provides a reason to accept these costs by connecting improvement efforts with service recipients, organizational effectiveness, and broader public value. Recent public-sector evidence supports this motivational pathway. Using data from Korean central-government public servants, Lee and Kim (2024) found that public service motivation was positively associated with innovative behavior in public organizations.

Employees with stronger PSM are more likely to view administrative and service improvements as meaningful opportunities to advance public purposes rather than as unrewarded additions to their formal duties. Research links PSM with discretionary citizenship behavior among public employees and shows that its behavioral effects become stronger when employees can see how their work benefits society (Van Loon et al., 2018). More directly, evidence among Chinese civil servants associates PSM with the generation and implementation of innovative improvements (Miao et al., 2018). PSM does not remove the need for discretion, organizational support, or implementation opportunities. It does, however, increase employees' willingness to invest effort in innovations whose primary benefits accrue to service users, the organization, or the wider public.

Based on the preceding arguments, we propose the following hypothesis:

**Hypothesis 4 (H4):** Public service motivation is positively associated with public servants' innovative behavior.

#### 2.3.3 Sequential mediation

Taken together, the preceding relationships suggest that psychological contract and PSM are theoretically positioned as sequential rather than independent mechanisms. Psychological contract is positioned first because it reflects employees' relatively proximal assessment of how the organization values their contributions and fulfills reciprocal obligations. Employees form and revise these beliefs through their everyday organizational experiences, particularly through the conduct of immediate supervisors who act as organizational agents and implement personnel practices on the organization's behalf (Op De Beeck et al., 2017; Kiefer et al., 2022). Inclusive leadership may therefore be associated with public servants' understanding of the employment relationship, which provides a relational basis for the workplace expression of public service motivation.

A favorable psychological contract can, in turn, provide a more credible organizational context in which public servants express their existing public service motives. When public servants perceive mutuality between their contributions and the organization's obligations, public-oriented effort is less likely to be experienced as a one-sided commitment, and the organization is more likely to be viewed as a viable vehicle for pursuing public purposes (Dabos and Rousseau, 2004). Although PSM has relatively enduring foundations, its salience and workplace expression can be reinforced or constrained by leadership, socialization, and HRM conditions (Hameduddin and Engbers, 2022; Piatak et al., 2020; Kjeldsen and Jacobsen, 2013). Psychological contract perceptions do not create public servants' public service values; rather, they may strengthen the extent to which public servants are willing to enact those values through their current organizational roles.

PSM provides the motivational direction for discretionary effort. Public servants with stronger PSM are more willing to undertake activities whose benefits accrue to service users, public organizations, or society, even when such activities require additional effort or offer uncertain personal returns (Van Loon et al., 2018). In particular, evidence among civil servants associates PSM with the generation and implementation of work-related improvements (Miao et al., 2018). Taken together, these relationships connect public servants' relational interpretation of inclusive supervisory conduct with a public-service motivational orientation associated with innovative behavior.

Based on the preceding arguments, we propose the following hypothesis:

**Hypothesis 5 (H5):** Psychological contract and public service motivation sequentially mediate the positive relationship between inclusive leadership and public servants' innovative behavior.

### 2.4 Supervisory interaction pressure as a same-source relational demand

The pathway developed above assumes that employees can approach and use the support offered by an inclusive supervisor. In public organizations, however, the same supervisor also sets expectations, evaluates performance, and enforces formal requirements (Op De Beeck et al., 2017; Lin and Kellough, 2019; Hill and Plimmer, 2024). Employees therefore encounter support and authority through the same relationship.

We define supervisory interaction pressure (SIP) as the psychological pressure public servants experience during direct interaction with their immediate supervisor. SIP is relationship-specific because it concerns encounters with a particular authority figure rather than the general intensity of work. Psychologically pressuring supervisory interaction may heighten vigilance about evaluation and make public servants more reluctant to raise uncertain ideas, disclose difficulties, or seek assistance from the supervisor.

SIP is related to, but distinct from, broader features of the work environment. Psychological safety concerns whether interpersonal risk taking is generally regarded as safe, whereas performance pressure concerns the urgency attached to demanding standards or consequential failure (Frazier et al., 2017; Edmondson and Bransby, 2023; Mitchell et al., 2018). SIP focuses more narrowly on the pressure attached to direct contact with a particular supervisor, and it does not require sustained hostile behavior (Tepper, 2000). An employee may therefore regard a team as generally safe and a supervisor as inclusive, yet still find direct supervisory encounters demanding because that supervisor controls evaluation and access to organizational resources.

#### 2.4.1 A relational contingency in the use and interpretation of supervisory resources

JD-R research increasingly recognizes leadership as intertwined with the demands and resources public servants encounter at work (Bakker and Demerouti, 2017; Tummers and Bakker, 2021). This perspective provides a basis for examining how the quality of supervisory interaction shapes the value of supportive leadership. In the present model, supervisory interaction pressure is expected to condition how public servants use and interpret inclusive conduct from the same supervisor. The relational value of inclusive leadership may therefore depend not only on the support supervisors provide, but also on the psychological conditions under which public servants approach and interpret that support.

This relational contingency may operate in two ways. First, interaction pressure may make supervisory resources more difficult to use. When approaching a supervisor feels psychologically pressuring, public servants may become more vigilant during the interaction or more reluctant to raise incomplete ideas, disclose problems, or request assistance from the person who controls relevant resources (Detert and Edmondson, 2011; Bolino et al., 2016). Second, interaction pressure may shape the relational meaning public servants assign to inclusive conduct. Public servants infer organizational intentions from recurring managerial behavior, and supportive signals are more credible when they are embedded in consistent patterns of treatment (Nishii et al., 2008; Bowen and Ostroff, 2004). Because supervisors often act as organizational representatives, psychologically pressuring interaction with the same supervisor may weaken openness and accessibility as signals of organizational recognition and reciprocity (Shoss et al., 2012).

The same-source configuration therefore supports two distinct moderation arguments. SIP can make it harder for employees to use the resources provided by inclusive leadership, and it can make inclusive conduct a less credible signal of organizational reciprocity. The first proposed mechanism concerns resource use; the second concerns how employees interpret the supervisor's behavior.

#### 2.4.2 Supervisory interaction pressure and the inclusive leadership-innovative behavior relationship

Inclusive leadership supports innovation by making the supervisor available for information, assistance, authorization, and feedback. These resources matter because innovative behavior requires employees to question established routines, promote uncertain proposals, and persist through implementation (Scott and Bruce, 1994; Yuan and Woodman, 2010). Managerial openness also gives employees a channel through which to raise improvement-oriented ideas and seek support for acting on them (Nembhard and Edmondson, 2006; Detert and Burris, 2007).

When SIP is low, public servants can approach an inclusive supervisor without devoting substantial attention to managing the encounter. They are better able to discuss work problems, test incomplete ideas, ask for assistance, and seek approval for implementation. This access is especially important in public organizations, where employee-led improvements often depend on supervisory coordination and formal authorization (Fernandez and Moldogaziev, 2013).

When SIP is high, public servants must devote more attention to self-presentation, emotion regulation, and the possibility of unfavorable evaluation. They may withhold concerns or tentative proposals and avoid seeking help from the person who controls key implementation resources (Morrison, 2022; Bolino et al., 2016). These demands consume capacity that could otherwise support idea development and implementation (Bakker et al., 2023). SIP should therefore make it harder for employees to turn the support and opportunities provided by inclusive leadership into innovative behavior.

Based on the preceding arguments, we propose the following hypothesis:

**Hypothesis 6 (H6):** Supervisory interaction pressure negatively moderates the relationship between inclusive leadership and public servants' innovative behavior, such that the positive relationship is weaker when supervisory interaction pressure is higher.

#### 2.4.3 Supervisory interaction pressure and the inclusive leadership-psychological contract relationship

Inclusive leadership can strengthen psychological contract when employees interpret supervisory conduct as evidence of organizational recognition and reciprocity. Immediate supervisors implement personnel practices, allocate opportunities, and communicate expectations on the organization's behalf (Op De Beeck et al., 2017). Employees may therefore treat their conduct as evidence of organizational intent, particularly when the supervisor is seen as a credible representative of the organization (Eisenberger et al., 2010). Openness, accessibility, and constructive responses can signal that employee contributions are valued and that reciprocal obligations will be honored (Dabos and Rousseau, 2004).

The strength of this signal depends on how well it fits employees' broader experience of the same supervisor. Employees infer managerial intentions from recurring patterns rather than isolated favorable acts, and consistent practices make organizational messages easier to interpret (Bowen and Ostroff, 2004). Recent public-sector evidence also shows that psychological contract fulfillment responds to employees' experience of organizational change and its management (Pieters et al., 2026). When SIP is low, inclusive conduct is consistent with an interaction that employees can approach without substantial vigilance, making the signal of recognition and reciprocity more credible.

High SIP creates a different interpretive context. The supervisor may invite participation while also making direct interaction feel evaluative or demanding. Because both cues come from the same person, employees cannot readily attribute the pressure to some other part of the work environment. This inconsistency weakens confidence that inclusive conduct reflects a durable organizational commitment rather than a conditional or closely monitored opportunity. Research on behavioral integrity and ambivalent leader relationships similarly shows that inconsistent experiences reduce leadership credibility and can produce simultaneous positive and negative judgments of the same supervisor (Lee et al., 2019; Simons, 2002). This problem is especially relevant in public performance management, where supervisors must support employee development while appraising performance and maintaining accountability (Lin and Kellough, 2019; Hill and Plimmer, 2024). SIP should therefore weaken the positive relationship between inclusive leadership and psychological contract.

Based on the preceding arguments, we propose the following hypothesis:

**Hypothesis 7 (H7):** Supervisory interaction pressure negatively moderates the relationship between inclusive leadership and public servants' psychological contract, such that the positive relationship is weaker when supervisory interaction pressure is higher.

#### 2.4.4 Conditional sequential mediation

H7 implies that SIP should influence the broader pathway linking inclusive leadership with innovative behavior through psychological contract and PSM. When SIP is lower, inclusive leadership should provide a stronger relational basis for favorable psychological contract perceptions. A more favorable psychological contract, in turn, provides a more supportive organizational context for the expression of public service motivation, which is associated with innovative behavior. When SIP is high, the weaker inclusive leadership–psychological contract relationship should reduce the strength of this sequential pathway.

Accordingly, supervisory interaction pressure is expected to condition not only the direct association between inclusive leadership and innovative behavior, but also the indirect pathway linking inclusive leadership with innovative behavior through psychological contract and PSM. Accordingly, we propose the following hypothesis:

**Hypothesis 8 (H8):** The positive indirect relationship between inclusive leadership and public servants' innovative behavior through psychological contract and public service motivation is weaker when supervisory interaction pressure is higher.

## 3 Data and methods

### 3.1 Research setting, sample, and procedure

Data were collected from tax civil servants across the tax administration system of S Province, China. The system included public servants working in general administration, tax collection administration, taxpayer services, tax inspection, and information technology. Immediate supervisors communicated work expectations, coordinated task and policy implementation, provided feedback, inspected work progress, and exercised formal administrative authority. These features describe the organizational setting and occupational scope of the study; their theoretical relevance was developed in the preceding sections.

The survey was administered between September and October 2023 through a third-party online platform. With assistance from the provincial tax administration, the survey invitation was distributed system-wide to eligible tax civil servants throughout the province. Participation was voluntary, and informed consent was obtained before respondents began the questionnaire. Participants accessed the survey link through their personal electronic devices and completed the questionnaire independently. Although the invitation procedure provided system-wide coverage, participation ultimately depended on public servants' willingness to respond. The study should therefore be understood as a system-wide voluntary survey rather than a probability sample or a census of completed responses. Moreover, it was administered anonymously and did not collect identifiers for immediate supervisors, work units, branches, or departments. Respondents therefore could not be linked to shared supervisors or organizational units.

The questionnaire incorporated procedural separation among the focal measures. Inclusive leadership, psychological contract, public service motivation, innovative behavior, and supervisory interaction pressure were presented in separate sections, with each construct appearing on an independent page. Respondents were required to answer and confirm all items on the current page before proceeding to the next section. This page-level separation was intended to create psychological distance among the measures, reduce immediate consistency cues, and make the complete theoretical model less apparent to respondents. These procedures cannot, however, eliminate common method concerns associated with cross-sectional self-report data (Podsakoff et al., 2003).

The online platform generated 9,379 survey records. Of these, 6,837 contained complete responses on the five focal variables: inclusive leadership, psychological contract, public service motivation, innovative behavior, and supervisory interaction pressure. Because the primary analyses also included gender, age, education, years of service, and organizational function as control variables, respondents with missing data on any study variable were excluded. This yielded a strict complete-case analytic sample of 4,511 tax civil servants. Missing values were not imputed in the primary analyses.

To assess whether this restriction introduced substantial selection on the focal constructs, we compared the retained complete cases with the focal-variable-complete observations excluded because of missing control data. Differences on the five focal constructs were small in magnitude, with absolute standardized mean differences ranging from 0.024 to 0.144. Sensitivity analyses using alternative treatments of years of service and missing control-variable data produced substantively similar focal estimates.

Table 1 reports the demographic and organizational characteristics of the final analytic sample. The sample included 2,181 men (48.3%) and 2,330 women (51.7%), with a mean age of 41.72 years (*SD* = 11.49). Most respondents held a bachelor's degree or above (89.8%). Respondents also had substantial years of service on average (*M* = 237.86 months, *SD* = 165.95), indicating an experienced public-sector sample. The largest organizational-function group was tax collection administration (41.2%), followed by general administration (36.0%), taxpayer services (13.2%), tax inspection (8.6%), and information technology (0.9%).

**Table 1:** Demographic and organizational characteristics of respondents (*N* = 4,511).

Characteristic	Category	*n*/*M*	%/*SD*
Gender	Male	2,181	48.30%
	Female	2,330	51.70%
Age	Mean (*SD*)	41.72	11.49
Education	Associate degree or below	458	10.20%
	Bachelor's degree or above	4,053	89.80%
Years of service	Mean months (*SD*)	237.86	165.95
Organizational function	General administration	1,624	36.00%
	Tax collection administration	1,858	41.20%
	Taxpayer services	597	13.20%
	Tax inspection	390	8.60%
	Information technology	42	0.90%

Percentages may not sum to 100 due to rounding.

### 3.2 Measures

All multi-item constructs were measured using scales adapted from prior research. Because the survey was administered in Chinese, the original measures were translated and adapted to the work context of tax civil servants. A translation and back-translation procedure was used to assess semantic equivalence across the original and Chinese versions (Brislin, 1970). One bilingual research team translated the original items into Chinese, and a separate bilingual team translated the Chinese items back into English. The two versions were compared, and discrepancies were discussed and revised to preserve the substantive meaning of the original constructs while ensuring that the wording was appropriate for the administrative and public service context. Unless otherwise indicated, respondents rated the items on five-point Likert-type scales ranging from 1 = strongly disagree to 5 = strongly agree. Higher scores indicated higher levels of the respective constructs. The complete wording of the measurement items is provided in Appendix A.

#### 3.2.1 Inclusive leadership

Inclusive leadership was measured using four items adapted from Carmeli et al. (2010). The items assessed the extent to which immediate supervisors were open, accessible, responsive, and supportive in work-related interactions. Representative content concerned whether supervisors encouraged collaboration, remained available when support was needed, and responded constructively to respondent contributions. The four-item scale demonstrated high internal consistency in the present study (Cronbach's α = 0.892).

#### 3.2.2 Psychological contract

Psychological contract was measured using four items adapted from Dabos and Rousseau (2004). The items assessed respondents' perceptions of the organization's returns, pride in organizational membership, occupational achievement, and future career development prospects. The wording was adapted to reflect the employment relationship experienced by tax civil servants. Higher scores represented a more positive perception of the reciprocal relationship between the respondent and the organization. The scale demonstrated good internal consistency (Cronbach's α = 0.865). Accordingly, the measure operationalized psychological contract as respondents' perceived positive exchange relationship with the organization, rather than as contract breach or a comprehensive assessment of all psychological-contract dimensions.

#### 3.2.3 Public service motivation

Public service motivation was measured using five items based on the global measure used by Wright et al. (2012). The items assessed the personal importance respondents attached to public service and their willingness to contribute to the public interest through their work. The measure was adapted to the tax administration context by referring, where appropriate, to serving taxpayers, contributing to the public interest through tax services, and providing services to local residents. These adaptations made the public-service referent concrete without changing the measure into an assessment of organizational attachment, job satisfaction, or anticipated personal reward. The scale demonstrated good internal consistency (Cronbach's α = 0.877).

#### 3.2.4 Innovative behavior

Innovative behavior was measured using four items adapted from Scott and Bruce (1994). The items assessed respondents' involvement in generating, promoting, and implementing new ideas in their work. Representative content concerned searching for improved processes, techniques, or methods, developing plans for implementing ideas, and seeking the resources needed to put those ideas into practice. The scale demonstrated good internal consistency (Cronbach's α = 0.850). The measure captures respondents' self-reported engagement in innovation-related behavior rather than the objective quality, organizational adoption, or service outcomes of specific innovations. Accordingly, the findings should not be interpreted as direct evidence of realized organizational innovation performance.

#### 3.2.5 Supervisory interaction pressure

Supervisory interaction pressure was measured using a single item that asked respondents to indicate the degree of psychological pressure they felt when directly interacting with their immediate supervisor. Responses ranged from 1 = never nervous to 5 = very nervous. The item appeared within a broader survey concerning tax performance management, which included content related to supervisory evaluation, goal attainment, promotion, performance ranking, accountability, rewards and sanctions, and supervisory inspection of work performance. We therefore use the item as a narrowly defined, context-specific indicator of psychological pressure experienced in direct supervisor-employee interaction within a performance-management environment.

The measure does not represent a comprehensive assessment of performance pressure. It does not directly measure workload, time pressure, target intensity, promotion pressure, formal appraisal consequences, or the linkage of performance outcomes to rewards and sanctions. Separate survey items concerning promotion and performance-outcome linkage were not included in the focal theoretical model. The label supervisory interaction pressure was therefore selected to reflect the item's immediate-supervisor referent and to avoid assigning it a broader meaning than the measurement supports. Because SIP was assessed using one item, internal consistency could not be estimated. The indicator should not be interpreted as a validated multidimensional scale or as capturing the full domain of relational and performance demands. Its limited breadth and measurement precision are considered in the Discussion.

#### 3.2.6 Control variables

The analyses controlled for gender, age, education, years of service, and organizational function. Gender was coded as 0 = female and 1 = male, and age was measured in years. Education was represented as a binary indicator, with associate degree or below as the reference category and bachelor's degree or above coded as 1. Years of service was measured in months and entered as a continuous variable in the primary analyses. Organizational function comprised general administration, tax collection administration, taxpayer services, tax inspection, and information technology. Because these categories have no meaningful numerical order, organizational function was represented by four dummy variables, with tax collection administration as the reference category.

The control variables were included because demographic background, years of service, educational attainment, and organizational function may be associated with public servants' work experience and opportunities to engage in innovative behavior.

### 3.3 Analytical strategy

Confirmatory factor analysis was conducted using AMOS 28.0 to assess the discriminant validity of the four multi-item constructs: inclusive leadership, psychological contract, public service motivation, and innovative behavior. The hypothesized four-factor measurement model was compared with alternative measurement models using conventional model-fit indices. Descriptive statistics, reliability coefficients, and correlations among the focal variables were then calculated. Because supervisory interaction pressure was measured using a single item, it was included as an observed variable rather than as a latent factor in the confirmatory factor analysis. All primary analyses were conducted using the strict complete-case analytic sample (*N* = 4,511).

The hypotheses were tested using the PROCESS macro developed by Hayes (2022). First, PROCESS Model 6 was used to examine the proposed ordered indirect pathway. Inclusive leadership was specified as the independent variable, psychological contract as the first mediator, public service motivation as the second mediator, and innovative behavior as the dependent variable. This analysis estimated the associations proposed in Hypotheses 1–4 and the ordered indirect association proposed in Hypothesis 5. Given the cross-sectional design, the analysis was interpreted as testing whether the observed associations were consistent with the proposed theoretical sequence rather than as establishing temporal or causal mediation.

Second, PROCESS Model 86 was used to examine the moderating role of supervisory interaction pressure. Supervisory interaction pressure was specified as the moderator of both the relationship between inclusive leadership and psychological contract and the direct relationship between inclusive leadership and innovative behavior. Model 86 was therefore used to test Hypotheses 6 and 7, as well as the conditional ordered indirect association proposed in Hypothesis 8. Supervisory interaction pressure was not specified as a moderator of the relationship between psychological contract and public service motivation or of the relationship between public service motivation and innovative behavior. This specification located the proposed boundary condition at the initial stage of the relational-to-public-service pathway and at the direct inclusive leadership–innovative behavior link.

Inclusive leadership and supervisory interaction pressure were mean-centered before their interaction term was constructed. Significant interaction effects were examined using simple-slope analyses at one standard deviation below and above the mean of supervisory interaction pressure. Indirect and conditional indirect associations were evaluated using bootstrap confidence intervals based on 5,000 resamples. An indirect or conditional indirect association was considered statistically supported when its 95% bootstrap confidence interval did not include zero. The same control variables specified in Section 3.2 were included throughout the hypothesis-testing analyses.

Because the focal variables were collected through the same questionnaire, two diagnostic procedures were used to examine the extent to which common method variance might influence the observed covariance among the measures. Harman's single-factor test was used as a descriptive diagnostic rather than as a definitive test of common method bias. In addition, the hypothesized measurement model was compared with a model incorporating a common method factor, and changes in model fit and the substantive measurement pattern were examined. These procedures were used to identify indications of a dominant common method component; they cannot establish that common method variance was absent. The measurement-model and common-method diagnostic results are reported before the hypothesis tests.

Two sensitivity analyses were conducted to assess the robustness of the focal estimates. First, years of service was alternatively represented using categorical indicators rather than continuous months. Second, missing values in the control variables were addressed using multiple imputation in the focal-variable-complete sample. The focal theoretical variables were not imputed. These analyses examined whether the main findings were sensitive to alternative treatments of years of service and missing control-variable data.

## 4 Results

### 4.1 Measurement validity and common method bias

Confirmatory factor analysis was conducted to assess the discriminant validity of the four multi-item constructs: inclusive leadership, psychological contract, public service motivation, and innovative behavior. As shown in Table 2, the hypothesized four-factor model demonstrated acceptable fit to the data, with RMSEA = 0.065, SRMR = 0.045, CFI = 0.954, and TLI = 0.945. Although the normed chi-square value was relatively large (χ^2^/df = 19.992), this result should be interpreted in light of the large sample size.

**Table 2:** Results of confirmatory factor analysis and CMV diagnostics (*N* = 4,511).

Model	χ^2^/*df*	RMSEA	SRMR	CFI	TLI
Four-factor model	19.992	0.065	0.045	0.954	0.945
Three-factor model a	70.651	0.124	0.070	0.827	0.798
Three-factor model b	63.134	0.117	0.085	0.846	0.819
Three-factor model c	66.374	0.120	0.087	0.838	0.810
Two-factor model	98.316	0.147	0.105	0.755	0.717
One-factor model	127.243	0.167	0.105	0.679	0.633
Four-factor model + common method factor	14.522	0.055	0.030	0.972	0.961

RMSEA, root mean square error of approximation; SRMR, standardized root mean square residual; CFI, comparative fit index; TLI, tucker-lewis index.

The four-factor model includes inclusive leadership, psychological contract, public service motivation, and innovative behavior as separate latent constructs. Model a combines inclusive leadership and public service motivation; Model b combines public service motivation and innovative behavior; Model c combines psychological contract and innovative behavior, psychological contract and public service motivation, and inclusive leadership and innovative behavior.

The hypothesized four-factor model fit the data substantially better than the alternative three-factor, two-factor, and one-factor models. These results support the empirical distinctiveness of inclusive leadership, psychological contract, public service motivation, and innovative behavior.

Because all focal variables were collected through a self-report questionnaire, common method bias was assessed using two approaches. First, Harman's single-factor test showed that the first unrotated factor accounted for 47.391% of the total variance, below the commonly used threshold of 50%. Second, a common method factor was added to the confirmatory factor analysis model. Compared with the original four-factor model, the changes in model fit were modest (Δ*CFI* = 0.018, Δ*TLI* = 0.016, Δ*RMSEA* = 0.010, and Δ*SRMR* = 0.015). Taken together, these diagnostics provided no evidence of a dominant common method factor. Nevertheless, given the single-source, cross-sectional design, some degree of common method variance and self-report bias cannot be excluded.

### 4.2 Descriptive statistics and correlations

Table 3 presents the means, standard deviations, correlations, and reliability coefficients for the focal variables. Inclusive leadership was positively correlated with psychological contract (*r* = 0.667, *p* < 0.01), public service motivation (*r* = 0.557, *p* < 0.01), and innovative behavior (*r* = 0.399, *p* < 0.01). Psychological contract was positively correlated with public service motivation (*r* = 0.635, *p* < 0.01) and innovative behavior (*r* = 0.405, *p* < 0.01). Public service motivation was also positively correlated with innovative behavior (*r* = 0.440, *p* < 0.01). These correlations were consistent with the expected positive relationships among inclusive leadership, the proposed relational and motivational mechanisms, and innovative behavior.

**Table 3:** Means, standard deviations, correlations, and reliability coefficients.

Variable	*M*	*SD*	1	2	3	4	5
1. *IL*	3.632	0.714	(0.898)				
2. *PC*	3.203	0.857	0.667^**^	(0.867)			
3. *PSM*	3.531	0.784	0.557^**^	0.635^**^	(0.872)		
4. *IB*	3.625	0.625	0.399^**^	0.405^**^	0.440^**^	(0.846)	
5. *SIP*	2.641	0.977	−0.079^**^	−0.121^**^	−0.091^**^	−0.107^**^	–

*N* = 4,511. *IL*, inclusive leadership; *PC*, psychological contract; *PSM*, public service motivation; *IB*, innovative behavior; *SIP*, supervisory interaction pressure. Cronbach's alpha coefficients are shown on the diagonal in parentheses. ^**^p < 0.01.

Supervisory interaction pressure showed small negative correlations with inclusive leadership (*r* = −0.079, *p* < 0.01), psychological contract (*r* = −0.121, *p* < 0.01), public service motivation (*r* = −0.091, *p* < 0.01), and innovative behavior (*r* = −0.107, *p* < 0.01). The hypothesized direct, indirect, and moderating relationships were examined more formally in the subsequent analyses.

### 4.3 Direct relationships

Table 4 reports the path estimates from the sequential mediation model using the strict complete-case analytic sample. Inclusive leadership was positively associated with innovative behavior (*b* = 0.1469, *SE* = 0.0162, *p* < 0.001), supporting Hypothesis 1. Inclusive leadership was also positively associated with psychological contract (*b* = 0.8156, *SE* = 0.0133, *p* < 0.001), supporting Hypothesis 2.

**Table 4:** Path Estimates for the Sequential Mediation Model.

Variable	*PC*	*PSM*	*IB*
Control variables			
Gender	0.0245	0.0452^*^	0.0905^***^
Age	0.0006	−0.0003	0.0003
Education	−0.0564	−0.0121	0.0325
Years of service	0.0005^***^	0.0006^***^	−0.0001
General administration	0.0277	0.0465^*^	0.0326
Taxpayer services	0.1557^***^	0.0743^**^	−0.0349
Tax inspection	0.0187	0.0441	0.0418
Information technology	0.1654	−0.0960	−0.0642
Independent variable			
IL	0.8156^***^	0.3037^***^	0.1469^***^
Mediators			
PC	–	0.4090^***^	0.0922^***^
PSM	–	–	0.2109^***^
*R* ^2^	0.4597	0.4548	0.2410
*F*	425.50^***^	375.38^***^	129.84^***^

*N* = 4,511. Values are unstandardized regression coefficients. Education was coded as 0 = associate degree or below and 1 = bachelor's degree or above. Tax collection administration was the reference category for organizational function. ^*^p < 0.05, ^**^p < 0.01, ^***^p < 0.001.

Psychological contract was positively associated with public service motivation after inclusive leadership and the control variables were taken into account (*b* = 0.4090, *SE* = 0.0137, *p* < 0.001), supporting Hypothesis 3. Public service motivation, in turn, was positively associated with innovative behavior after accounting for inclusive leadership, psychological contract, and the control variables (*b* = 0.2109, *SE* = 0.0140, *p* < 0.001), supporting Hypothesis 4.

The model also retained the shorter associations incorporated in the proposed sequential model. Inclusive leadership was positively associated with public service motivation (*b* = 0.3037, *SE* = 0.0166, *p* < 0.001), and psychological contract was positively associated with innovative behavior (*b* = 0.0922, *SE* = 0.0141, *p* < 0.001). These results are consistent with the proposed ordered indirect pattern while also indicating the presence of shorter relational and motivational associations.

### 4.4 Indirect and sequential indirect effects

Indirect effects were tested using bootstrap confidence intervals based on 5,000 resamples. As shown in Table 5, the total effect of inclusive leadership on innovative behavior was positive and statistically significant [*b* = 0.3566, *BootSE* = 0.0168, 95% *CI* (0.3241, 0.3898)]. The direct effect remained significant after psychological contract and public service motivation were included in the model [*b* = 0.1469, *BootSE* = 0.0221, 95% *CI* (0.1041, 0.1904)]. The total indirect effect was also significant [*b* = 0.2096, *BootSE* = 0.0150, 95% *CI* (0.1805, 0.2393)].

**Table 5:** Bootstrap results for indirect and sequential indirect effects.

Effect	Estimate	*BootSE*	Boot LLCI	Boot ULCI	Proportion of total effect
Total effect	0.3566	0.0168	0.3241	0.3898	–
Direct effect	0.1469	0.0221	0.1041	0.1904	41.2%
Total indirect effect	0.2096	0.0150	0.1805	0.2393	58.8%
Ind1: *IL*→*PC*→*IB*	0.0752	0.0151	0.0457	0.1043	21.1%
Ind2: *IL*→*PSM*→*IB*	0.0641	0.0073	0.0502	0.0791	18.0%
Ind3: *IL*→*PC*→*PSM*→*IB*	0.0704	0.0072	0.0563	0.0848	19.7%

*N* = 4,511. Bootstrap confidence intervals are based on 5,000 resamples. *IL*, inclusive leadership; *PC*, psychological contract; *PSM*, public service motivation; *IB*, innovative behavior; *LLCI*, lower limit confidence interval; *ULCI*, upper limit confidence interval. An indirect effect is significant when the confidence interval does not include zero.

Most importantly, the ordered sequential indirect association of inclusive leadership with innovative behavior through psychological contract and public service motivation was positive and statistically significant [*b* = 0.0704, *BootSE* = 0.0072, 95% *CI* (0.0563, 0.0848)]. Because the bootstrap confidence interval did not include zero, Hypothesis 5 was supported. This finding was consistent with the proposed relational-to-public-service sequence in which inclusive leadership was associated with a more positive psychological contract, psychological contract was associated with stronger public service motivation, and public service motivation was associated with innovative behavior. Given the cross-sectional design, this ordered indirect association should not be interpreted as establishing temporal or causal mediation.

The two shorter indirect effects were also significant. The indirect effect through psychological contract alone was *b* = 0.0752 [*BootSE* = 0.0151, 95% *CI* (0.0457, 0.1043)], while the indirect effect through public service motivation alone was *b* = 0.0641 [*BootSE* = 0.0073, 95% *CI* (0.0502, 0.0791)]. Together, these results were consistent with the proposed ordered indirect sequence while also showing statistically supported shorter indirect associations.

### 4.5 Moderating Effects of Supervisory Interaction Pressure

Table 6 reports the moderating effects of supervisory interaction pressure. The interaction between inclusive leadership and supervisory interaction pressure was negatively associated with innovative behavior (*b* = −0.0524, *SE* = 0.0096, *p* < 0.001). Simple slope analysis showed that the positive relationship between inclusive leadership and innovative behavior was stronger when supervisory interaction pressure was lower (*b* = 0.1958, *SE* = 0.0188, *p* < 0.001) than when supervisory interaction pressure was higher (*b* = 0.0936, *SE* = 0.0185, *p* < 0.001). Hypothesis 6 was therefore supported.

**Table 6:** Moderating effects of supervisory interaction pressure.

Moderated relationship	Interaction coefficient	SE	*P*	Effect at low SIP	Effect at high SIP	Conclusion
*IL* × *SIP*→*IB*(H6)	−0.0524	0.0096	< 0.001	0.1958^***^	0.0936^***^	Supported
*IL* × *SIP*→*PC*(H7)	−0.0236	0.0111	0.034	0.8318^***^	0.7857^***^	Supported

*N* = 4,511. Low and high *SIP* refer to values one standard deviation below and above the mean of supervisory interaction pressure. Values for low and high *SIP* are simple slopes. ^*^p < 0.05, ^***^p < 0.001.

Inclusive leadership remained positively associated with innovative behavior at both levels of supervisory interaction pressure. However, the decrease in the simple slope from 0.1958 to 0.0936 indicates that the positive association between inclusive leadership and innovative behavior was attenuated when direct supervisory interaction was experienced as more psychologically pressuring.

Supervisory interaction pressure also moderated the relationship between inclusive leadership and psychological contract. The interaction term was negative and statistically significant (*b* = −0.0236, *SE* = 0.0111, *p* = 0.034). The positive relationship between inclusive leadership and psychological contract was stronger when supervisory interaction pressure was lower (*b* = 0.8318, *SE* = 0.0170, *p* < 0.001) than when it was higher (*b* = 0.7857, *SE* = 0.0174, *p* < 0.001). Hypothesis 7 was therefore supported. These simple-slope patterns are illustrated in Figure 2.

**Figure 2:**
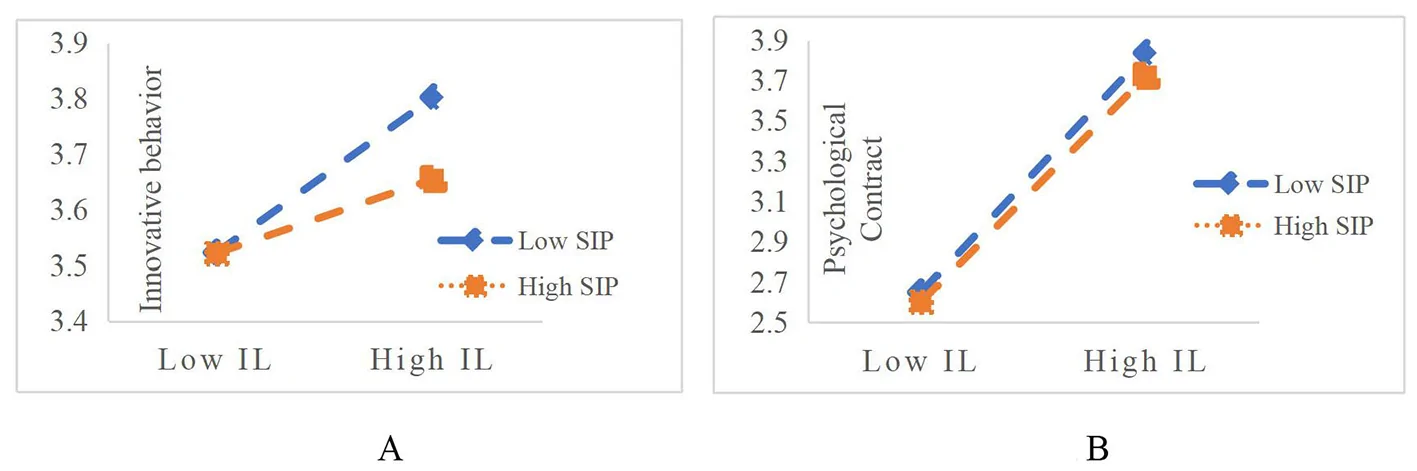
Simple slopes for the moderating effects of supervisory interaction pressure. **(A)** presents the inclusive leadership-innovative behavior relationship, and **(B)** presents the inclusive leadership-psychological contract relationship, at one standard deviation below and above the mean of supervisory interaction pressure.

Together, these results indicate that supervisory interaction pressure weakened, but did not reverse, the positive relationships between inclusive leadership and both psychological contract and innovative behavior.

### 4.6 Conditional sequential indirect effect

Table 7 reports the moderated mediation results. For the indirect relationship between inclusive leadership and innovative behavior through psychological contract, the index of moderated mediation was negative (index = −0.0021, *BootSE* = 0.0012), but the 95% bootstrap confidence interval included zero [−0.0046, 0.00004]. Thus, the weakening of this shorter indirect association was not statistically supported in the primary analysis.

**Table 7:** Moderated mediation results.

Indirect path	Index of moderated mediation	*BootSE*	Boot LLCI	Boot ULCI
*IL*→*PC*→*IB*	−0.0021	0.0012	−0.0046	0.00004
*IL*→*PC*→*PSM*→*IB* (H8)	−0.0020	0.0011	−0.0042	0.00004

*N* = 4,511. Bootstrap confidence intervals are based on 5,000 resamples. *LLCI*, lower limit of the confidence interval; *ULCI*, upper limit of the confidence interval. A moderated mediation effect is statistically supported when the confidence interval for the index of moderated mediation does not include zero.

For the ordered sequential indirect relationship through psychological contract and public service motivation, the index of moderated mediation was also negative (index = −0.0020, *BootSE* = 0.0011), but the 95% bootstrap confidence interval again included zero [−0.0042, 0.00004]. Hypothesis 8 was therefore not supported.

The negative point estimates were consistent with the theoretical expectation that supervisory interaction pressure would weaken indirect associations involving psychological contract. However, because the bootstrap confidence intervals included zero, the primary analysis did not provide sufficient statistical evidence that SIP significantly moderated the indirect or sequential indirect associations.

### 4.7 Sensitivity analyses

Sensitivity analyses produced substantively similar focal estimates. First, when years of service was represented using categorical indicators rather than continuous months, the principal mediation paths remained stable. The interaction between inclusive leadership and supervisory interaction pressure remained negative for innovative behavior (*b* = −0.0514, *p* < 0.001) and for psychological contract (*b* = −0.0261, *p* = 0.017).

Second, multiple-imputation analyses were conducted to examine whether the focal estimates were sensitive to missing control-variable data. Missing values were imputed only for the control variables; the focal theoretical variables were not imputed. The imputed-data estimates were substantively consistent with the complete-case results. The interaction between inclusive leadership and supervisory interaction pressure remained negative for innovative behavior [*b* = −0.0458, *SE* = 0.0075, *p* < 0.001, 95% *CI* (−0.0604, −0.0311)] and for psychological contract [*b* = −0.0262, *SE* = 0.0085, *p* = 0.002, 95% *CI* (−0.0429, −0.0095)]. The main focal-path estimates were also substantively similar to those obtained from the complete-case analysis.

## 5 Discussion

This study examined how inclusive leadership is associated with innovative behavior among civil servants working across a provincial tax administration system. Drawing on job demands-resources theory and integrating psychological contract and public service motivation research, the study considered both the resources provided by inclusive supervisors and the psychological pressure public servants may experience when interacting with the same supervisors.

The findings supported Hypotheses 1–7, with one important qualification. Inclusive leadership was positively associated with innovative behavior, both directly and through an ordered indirect association involving psychological contract and public service motivation. Supervisory interaction pressure weakened the positive relationships between inclusive leadership and both psychological contract and innovative behavior. However, the moderated mediation tests did not provide sufficient statistical evidence that SIP significantly weakened the indirect or ordered sequential indirect relationships; accordingly, Hypothesis 8 was not supported. Inclusive leadership nevertheless remained positively associated with both outcomes at higher levels of SIP, suggesting that interaction pressure constrained rather than eliminated the benefits of inclusive leadership.

### 5.1 Theoretical contributions

This study makes two contributions to public personnel management research. First, it develops a relationally contingent account of public-sector supervision by examining how the value of inclusive leadership depends on the supervisory interaction context in which it is experienced. Inclusive leadership research generally emphasizes supervisors' supportive functions, including inviting employee input, remaining accessible, and responding constructively to employee contributions (Alang et al., 2022; Ashikali, 2023). Public-sector supervisors, however, also monitor performance, communicate expectations, conduct work inspections, and exercise formal administrative authority (Op De Beeck et al., 2017; Vidè et al., 2023). Consistent with established JD-R research, we do not treat the coexistence of supervisory resources and demands as a novel theoretical claim (Bakker and Demerouti, 2017; Tummers and Bakker, 2021). Rather, the contribution lies in specifying a relationship-specific configuration in which psychological pressure attached to direct interaction with the same authority figure conditions the observed associations of inclusive leadership with relational and behavioral outcomes.

This same-source relational contingency matters for both behavioral and relational outcomes. Supervisory interaction pressure attenuated the positive association between inclusive leadership and innovative behavior, suggesting that public servants may be less able or willing to use available supervisory support when direct interaction requires greater vigilance and self-regulation. SIP also weakened the association between inclusive leadership and psychological contract, indicating that pressure from the same supervisor may reduce the credibility of openness and accessibility as signals of organizational recognition and reciprocity. These findings complement research on ambivalent and paradoxical leader relationships by showing how enabling and constraining experiences can coexist in routine public personnel management (Zhang et al., 2014; Lee et al., 2019). More broadly, they suggest that the value of inclusive leadership depends not only on the support supervisors offer, but also on the relational conditions under which public servants approach and interpret those supervisors. This pattern is particularly relevant in tax administration, where supervisors often control access to work information, procedural review, performance feedback, and approval for implementing changes. When direct interaction with supervisors feels psychologically pressuring, public servants may view requests for support or idea promotion as carrying evaluative risk. In such a context, inclusive conduct may remain beneficial, but its relational signal becomes less secure and its practical usefulness more constrained.

Second, the study develops a relational-to-public-service explanation of how inclusive leadership is associated with innovative behavior. Previous research has linked inclusive leadership with psychological safety, employee voice, empowerment, and workplace inclusion (Carmeli et al., 2010; Alang et al., 2022; Randel et al., 2017). The present study extends this literature by distinguishing two theoretically proposed mechanisms with different functions. Psychological contract captures public servants' assessment of mutuality and reciprocity in the employment relationship, whereas PSM captures the motivational orientation that directs discretionary effort toward public purposes (Dabos and Rousseau, 2004; Perry et al., 2010). The significant ordered indirect association is consistent with the argument that inclusive supervisory conduct may support innovation by strengthening a credible reciprocal relationship within which public servants can express their public service motives.

This interpretation does not imply that favorable psychological contract perceptions create public service values. PSM has relatively enduring foundations, but its salience and workplace expression can be reinforced or constrained by leadership, socialization, and HRM conditions (Hameduddin and Engbers, 2022; Piatak et al., 2020; Wright et al., 2012; Moynihan and Pandey, 2007). A reciprocal employment relationship may therefore make public servants more willing to enact existing public service motives through their current organizational roles. By connecting this relational appraisal with PSM, the study links a general employment-relationship mechanism to a motivation that is especially consequential in public organizations.

This contribution also extends public-sector inclusive leadership research from employees' willingness to participate or express opinions to their efforts to generate, promote, and implement work improvements. That extension is particularly relevant in regulated organizations such as tax administrations, where employee innovation must operate within legal requirements, hierarchical approval, and accountability arrangements. Employees may identify opportunities for improvement but still depend on supervisors for information, authorization, coordination, and implementation resources (Nguyen et al., 2023; Lee and Kim, 2024). The contribution is therefore not simply that inclusive leadership is positively associated with innovation. Rather, the findings are consistent with a theoretically proposed public personnel pathway linking supportive supervision with perceived reciprocity, public service motivation, and improvement-oriented behavior directed toward organizational and public purposes.

### 5.2 Practical implications

The findings offer three practical implications for public personnel management. First, public organizations should treat inclusive leadership as a concrete managerial capability for supporting innovation among public servants. Supervisors can encourage innovation by actively inviting employees to identify work problems, listening seriously to their suggestions, responding constructively to unsuccessful ideas, and providing timely feedback on whether proposed improvements can be adopted. Leadership development should therefore emphasize observable behaviors such as active listening, participatory problem solving, accessibility, and constructive feedback rather than relying only on general appeals for managers to become more supportive.

Second, public organizations should pay attention to how public servants experience direct interactions with their supervisors. Monitoring, work inspection, performance feedback, and managerial authority are necessary in public organizations, particularly in regulatory agencies. The findings do not suggest that these functions should be reduced or abandoned. Rather, supervisors should perform them in ways that avoid creating unnecessary psychological pressure. Clearlydistinguishing developmental discussions from formal evaluation, explaining the purpose of supervisory meetings, providing specific rather than personalized criticism, and allowing public servants to discuss difficulties can make interactions more predictable and constructive. Managers should also avoid sending inconsistent signals by inviting public servants to raise new ideas while responding defensively or critically when those ideas challenge existing practices.

Third, innovation management should strengthen both organizational reciprocity and the public-service meaning of employee contributions. Public servants are more likely to invest discretionary effort when they believe that the organization values their contributions and provides credible support. Public organizations should acknowledge improvement efforts, provide reasonable access to information and implementation resources, and communicate the outcomes of employee suggestions. Not every idea needs to be adopted, but public servants should receive a clear response rather than perceiving participation as symbolic.

Managers should also explain how proposed improvements contribute to public purposes. In tax administration, innovation may improve taxpayer services, administrative accuracy, procedural fairness, policy implementation, or the efficient use of public resources. Connecting innovation initiatives to these outcomes can help public servants understand how their efforts contribute to the public interest and are associated with concrete improvement behavior.

Overall, the results suggest that encouraging innovation requires more than adopting an inclusive leadership style. Public organizations must also ensure that public servants can interact with supervisors without excessive apprehension and that their contributions are accompanied by organizational support and a credible connection to public service outcomes.

### 5.3 Limitations and future research

Several limitations should be acknowledged. First, the cross-sectional, single-source, and self-reported design limits strong causal conclusions and creates potential risks of common method variance and self-report bias. Although procedural separation and statistical diagnostics were used to assess common method concerns, these procedures cannot eliminate them. Moreover, although the findings are consistent with the proposed theoretical sequence from inclusive leadership through psychological contract and public service motivation to innovative behavior, reverse or reciprocal relationships remain possible. Future research could use longitudinal and multisource designs, obtain supervisor or other-source ratings of innovative behavior, and, where feasible, incorporate objective or administrative indicators of implemented improvements or performance outcomes.

Second, the survey was administered anonymously and did not collect identifiers for immediate supervisors, work units, branches, or departments. Respondents therefore could not be linked to shared supervisors or organizational units. Accordingly, we could neither estimate the magnitude of supervisor- or team-level clustering nor adjust the analyses for potential dependence among respondents sharing the same supervisor or organizational unit. Future research with identifiable supervisory or organizational nesting structures could estimate such dependence directly and use multilevel or cluster-adjusted analyses where appropriate.

Third, supervisory interaction pressure was measured with a single item capturing the psychological pressure respondents experienced when interacting directly with their immediate supervisors. Although this measure fits the specific relational focus of the study, it cannot fully represent the different ways in which such pressure may be experienced and should therefore be interpreted as a narrow, context-specific indicator rather than a comprehensive measure of supervisory or performance-related pressure. Future research should develop and validate a multi-item scale of supervisory interaction pressure that can distinguish, for example, pressure associated with evaluation, supervisory authority, communication style, anticipated criticism, and accountability expectations.

Fourth, the primary analyses used a strict complete-case analytic sample because missing data were present in the control variables. Missing values were not imputed in the primary analyses. Although missingness diagnostics showed small differences between retained and excluded observations on the focal constructs, and sensitivity analyses using alternative treatments of years of service and missing control-variable data produced substantively similar focal estimates, complete-case selection remains a limitation. Future studies should collect complete demographic and organizational-function information at the outset or use designs that reduce missingness in control variables.

Despite these limitations, the study provides evidence from a large public-sector sample that inclusive leadership is positively associated with innovative behavior and that the observed indirect associations are consistent with the proposed relational-to-public-service sequence. Supervisory interaction pressure also attenuated key direct and relational associations involving inclusive leadership, although the hypothesized conditional sequential indirect association was not statistically supported.

## 6 Conclusion

This study examined how inclusive leadership is associated with innovative behavior among 4,511 tax civil servants across a provincial tax administration system in China. Inclusive leadership was positively associated with innovative behavior, both directly and through an ordered indirect association involving psychological contract and public service motivation. Supervisory interaction pressure weakened the positive relationships between inclusive leadership and both psychological contract and innovative behavior. However, the moderated mediation tests did not provide sufficient statistical evidence that supervisory interaction pressure weakened the indirect or ordered sequential indirect associations; accordingly, Hypothesis 8 was not supported.

These findings highlight the relational conditions under which inclusive leadership is experienced in public organizations. Supervisors can provide valuable resources through openness, accessibility, and responsiveness, while the findings suggest that psychological pressure attached to direct supervisory interaction may constrain the extent to which public servants benefit from those resources. For public personnel management, the implication is that developing inclusive leadership behavior may be insufficient when public servants continue to experience direct interactions with supervisors as psychologically pressuring. Public organizations should combine expectations for supervisory openness, accessibility, and responsiveness with interaction practices that enable public servants to raise problems, propose improvements, and seek support without unnecessary apprehension. By connecting supervisory resources, perceived mutuality and reciprocity, public service motivation, and supervisory interaction pressure, the findings suggest that public servants' innovative behavior is associated not only with supportive leader behavior but also with the relational conditions under which that behavior is experienced.

## Data Availability

The dataset for the study is available from the corresponding author upon reasonable request due to ethical restrictions.

## Appendix A

### Measurement items

Unless otherwise indicated, respondents rated the following items on a five-point Likert-type scale ranging from 1 = strongly disagree to 5 = strongly agree; the supervisory interaction pressure item was rated using response anchors from 1 = never nervous to 5 = very nervous. The items below are English translations of the Chinese questionnaire items used in the survey.

**Table A.1:** Measurement items

Construct	Item
Inclusive leadership	IL1. My supervisor creates a trusting work environment.
	IL2. My supervisor encourages open communication.
	IL3. My supervisor encourages collaboration with others in completing tasks.
	IL4. My supervisor handles work-related decisions in a fair manner.
Psychological contract	PC1. The returns provided by the organization are close to what I expected.
	PC2. As a member of this organization, I feel proud.
	PC3. I have a strong sense of achievement in my current occupation.
	PC4. I feel positive about my future career development prospects.
Public service motivation	PSM1. I am willing to serve taxpayers even without additional compensation.
	PSM2. I regard serving taxpayers as my duty.
	PSM3. I am willing to sacrifice my own interests for the benefit of taxpayers.
	PSM4. I am willing to work long term in grassroots departments to provide continuous public services to local residents.
	PSM5. Taxpayer satisfaction is very important to me.
Innovative behavior	IB1. To realize new ideas, I try to obtain the resources needed.
	IB2. To realize new ideas, I develop appropriate plans and arrangements.
	IB3. In my work, I always seek to apply new processes, techniques, or methods.
	IB4. Overall, I consider myself to be an innovative person.
Supervisory interaction pressure	SIP1. Based on your experience, how much psychological pressure do you feel when directly interacting with your immediate supervisor?
